# Epigenetic Control of Stress-Induced Depression: Emerging Roles of HDAC3 and HDAC6

**DOI:** 10.3390/ijms27167313

**Published:** 2026-08-16

**Authors:** Arathy S. Mohan, Narayanan Jayasankar, Vivekanand Ankush Kashid, Ravish J. Patel, Bhupendra Prajapati

**Affiliations:** 1 Department of Pharmacology, SRM College of Pharmacy, Faculty of Medicine and Health Sciences, SRM Institute of Science and Technology, Kattankulathur, Chengalpattu 603203, Tamil Nadu, India; as7439@srmist.edu.in; 2 MABD Institute of Pharmaceutical Education and Research, Babhulgaon, Yeola, Nashik 423401, Maharashtra, India; kashidvivek5@gmail.com; 3 Centre for Research Impact and Outcome, Chitkara College of Pharmacy, Chitkara University, Rajpura 140401, Punjab, India; 4 Ramanbhai Patel College of Pharmacy, Charotar University of Science and Technology, CHARUSAT Campus, Anand 388421, Gujarat, India; ravishpatel.ph@charusat.ac.in; 5 Department of Pharmaceutics, Parul Institute of Pharmacy, Faculty of Pharmacy, Parul University, Waghodia, Vadodara 391760, Gujarat, India; 6 Department of Industrial Pharmacy, Faculty of Pharmacy, Silpakorn University, Nakhon Pathom 73000, Thailand

**Keywords:** major depressive disorder, HDAC3, HDAC6, epigenetics, neuroplasticity, neuroinflammation, stress response, GR signaling, antidepressant therapy

## Abstract

Major depressive disorder (MDD) is a heterogeneous psychiatric disorder characterized by impaired mood, neuroplasticity, neuroinflammation, and dysregulated stress response systems. Chronic stress can induce epigenetic changes leading to depression. Evidence suggests that histone acetylation and deacetylation are epigenetic processes involved in changes in gene expression. Histone deacetylases (HDACs) modulate chromatin structure and transcription, and their dysregulation is associated with stress susceptibility, decreased brain-derived neurotrophic factor (BDNF) signaling, impaired synaptic plasticity, and inflammatory activation. HDAC isoforms HDAC3 and HDAC6 have emerged as epigenetic regulators in stress-induced depression. HDAC3 is a transcriptional regulator involved in neuroplasticity-related gene expression, inflammatory signaling, and glucocorticoid receptor-mediated stress responses. In contrast, HDAC6 is cytoplasmic and regulates non-histone substrates involved in microtubule dynamics, synaptic function, protein trafficking, and the regulation of the hypothalamic–pituitary–adrenal axis (HPA axis). Preclinical studies show that HDAC3 or HDAC6 inhibition can exert antidepressant-like effects by promoting neuroplasticity, reducing neuroinflammation, and restoring stress-related signaling. Dual targeting is an interesting therapeutic approach because both regulate complementary mechanisms. However, clinical translation is limited by poor blood–brain barrier penetration, systemic toxicity, insufficient isoform selectivity, and a lack of clinical evidence. This review summarizes the roles and mechanisms of HDAC3 and HDAC6, the rationale for dual targeting, translational limitations, and future therapeutic perspectives.

## 1. Introduction

Major depressive disorder (MDD) is a prevalent psychiatric illness characterized by the loss of interest in daily activities and persistent sadness-like symptoms for at least two weeks. Additional symptoms include changes in appetite, lack of energy, insomnia, lack of concentration, anxiety, restlessness, indecisiveness, worthlessness, guilt, hopelessness, self-harm, and suicidal thoughts. As reported by the World Health Organization (WHO), depression affects 4% of the global population, corresponding to approximately 332 million people worldwide. The prevalence of depression is estimated at 5.7% among adults (4.6% males, 6.9% females) and 5.9% among adults above the age of 70 years. Depression is approximately one and a half times higher in females than in males. Globally, more than 10% of women who either are pregnant or have recently given birth suffer from depression. In 2021, approximately 727,000 individuals died by suicide. Suicide is the third leading cause of death among adolescents and young adults aged between 15 and 29 years. MDD is considered to be one of the largest contributors to global disability and suicide, as the second largest cause of mortality among those aged 15–29 years, and accounts for approximately 800,000 deaths annually [1]. Antidepressants like serotonin and noradrenaline reuptake inhibitors are less effective since 30% of depressed people are pharmacoresistant. The genetic framework can combine with external adversity such as trauma, social conflict, and early-life neglect to cause long-lasting epigenetic changes associated with depression. Long-term exposure to various stressors modifies histone regulation and DNA methylation in important neurobiological pathways that underlie stress response and mood regulation [2].

Gene expression changes, known as epigenetic manipulations, are linked to alterations in chemical modifications of cytosine residues in DNA and histone proteins [3]. Histone deacetylases (HDACs) are a highly conserved protein family in all eukaryotes, and their primary function is to remove acetyl groups from DNA-binding histone proteins. This process is usually associated with specific repressive effects on gene expression and decreased chromatin accessibility for transcription factors (TFs) [4]. Each of the four major classes of 18 HDAC isoforms, I, II, III, and IV, has distinct biological roles and tissue distributions. Numerous cancer types, as well as neurological and inflammatory illnesses, have been connected to HDAC dysregulation [5]. Different HDAC inhibitors have been created based on the different biological activities of HDACs. Some of these inhibitors have been put on the market to treat specific types of cancer. Nonetheless, most have undergone evaluation in different stages of clinical studies [3].

Emerging evidence suggests that alterations in gene expression contribute to structural and functional changes in multiple brain areas and are implicated in the heterogeneity and pathogenesis of the illness. Epigenetic modifications that alter chromatin structure to regulate programs of gene expression have been linked with depression-like behavior, the effect of antidepressants, and resistance to depression or ‘resilience’ in animal models, with growing evidence for similar mechanisms occurring in the postmortem brains of depressed individuals [6]. In stress-related models of depression, lower histone acetylation in the hippocampus and prefrontal cortex is associated with the decreased expression of brain-derived neurotrophic factor (BDNF) and cAMP response element-binding protein (CREB), which are essential regulators of neuroplasticity and mood stabilization. Changes in histone modifications have also been linked to neuroinflammation and neuronal injury in depression [7]. Chronic stress increases HDAC activity in these brain regions, suppressing mood-related genes, while the pharmacological inhibition of HDACs can reverse these effects and produce antidepressant-like outcomes in animal models [8]. Among all the HDACs, HDAC3 is a key epigenetic regulator that maintains chromatin structure and regulates gene expression [9]. Another class IIb HDAC, HDAC6, is involved in memory formation and synaptic plasticity. It can modulate protein breakdown, control cytoskeletal dynamics, and alter gene expression [10]. Both HDAC3 and HDAC6 are important in depression due to their distinct roles in transcriptional regulation and cytoskeletal dynamics, respectively.

Research on dual HDAC3/HDAC6 inhibitors has predominantly concentrated on cancer therapy [11]; nevertheless, the synergistic functions of HDAC3 and HDAC6 in neuronal activity indicate their possible significance in neuropsychiatric disorders, including depression. This review summarizes the role of HDACs in depression, discusses HDAC inhibitors as emerging therapeutic candidates, and highlights HDAC3 and HDAC6 as mechanistically relevant isoforms in stress-induced depression.

## 2. Histone Deacetylases: Classification and Role in Depression

The family of enzymes known as HDACs is important for many biological processes, mostly because of their capacity to inhibit gene transcription [12]. There are four classes of HDACs: class I HDACs, which consist of HDAC1, 2, 3, and 8; class II HDACs, which consist of HDAC 4, 5, 6, 7, 9, and 10; class III HDACs, which comprise sirtuins (SIRTs); and class IV HDACs, comprising HDAC11 [13].

Histone acetylation promotes chromatin remodeling and transcriptional activation. It is controlled by histone acetyltransferases (HATs), which catalyze acetylation, and HDACs, which remove acetyl groups from lysine residues, primarily on histones H3 and H4, causing chromatin condensation and transcriptional repression. Increasing evidence suggests that abnormal epigenetic regulation, as defined by altered histone acetylation and HDAC expression in specific brain areas, contributes to the pathogenesis of mood disorders. Neurobiological alterations in histone acetylation and HDAC expression in the hippocampus have been identified in a variety of stress-induced depression animal models [14]. Neuroplasticity includes synaptic plasticity, neurogenesis, cell development, and remodeling, and depression is thought to result from decreased adaptive responses to environmental stress due to the dysfunction of these processes. Structural and molecular modifications identified in persons with depression validate this idea, whereas increased neuroplasticity, including neurogenesis, synaptogenesis, and dendritic branching, serves as a prevalent mechanism facilitating the effects of antidepressants. Consistent with this framework, alterations in the expression of neuroplasticity-related genes controlled by HDAC activity have been documented in major depressive disorder, including brain-derived neurotrophic factor (BDNF), synapsin 1 (Syn1), early growth response protein 1 (Egr1), and glycogen synthase kinase 3β (GSK3β). Reduced brain-derived neurotrophic factor (BDNF) expression, significantly regulated by HDACs, may contribute substantially to the onset and maintenance of depression [15]. Altered HDAC expression has long been associated with carcinogenesis, and more recent studies have linked this to depressive disorders [16].

**Figure 1:**
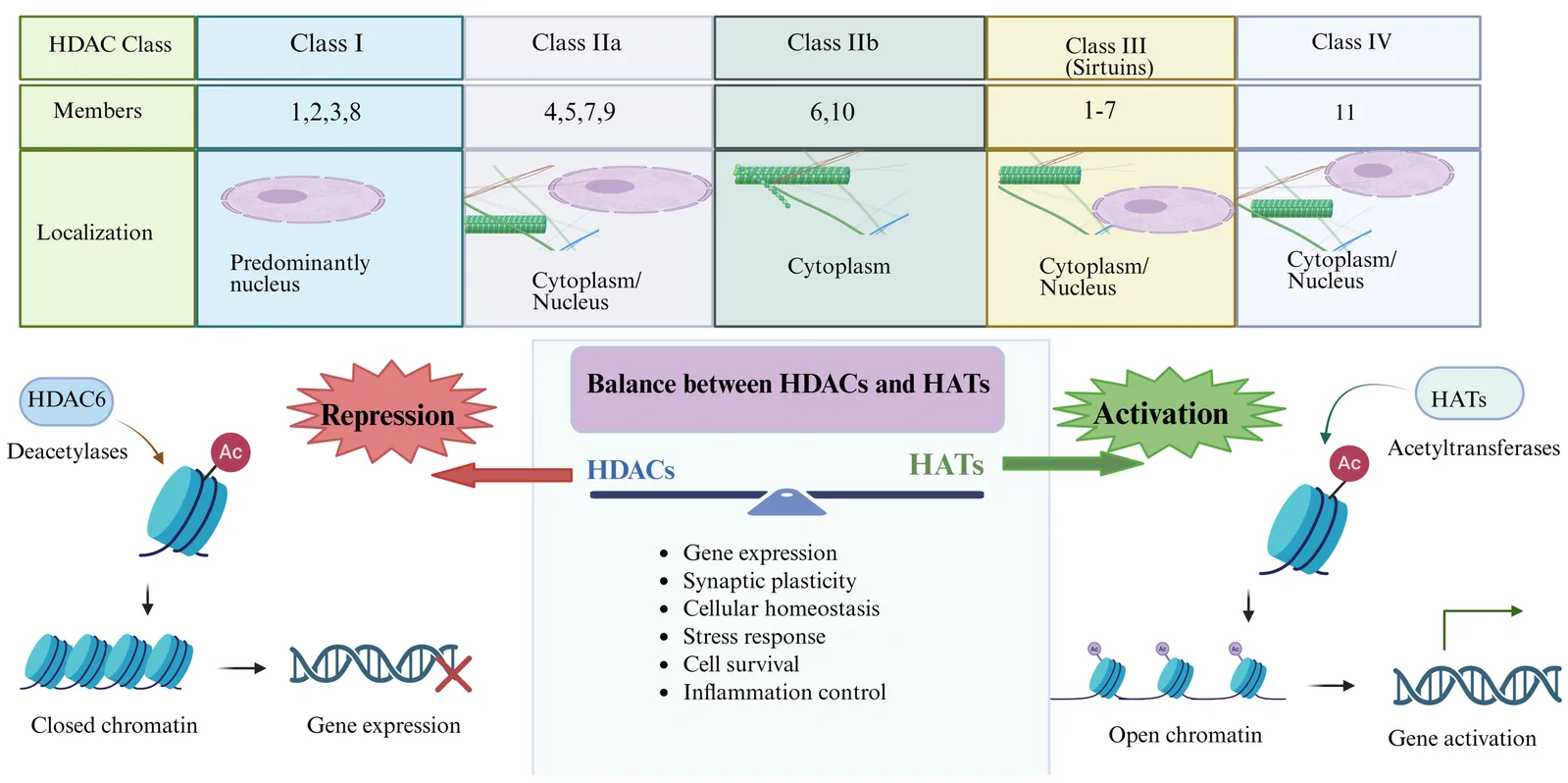
The classification of histone deacetylases (HDACs) and the role of HDAC3 in transcriptional regulation. Histone deacetylases (HDACs) are grouped into four classes based on their structure and subcellular localization. HDAC3-mediated histone deacetylation promotes chromatin condensation and transcriptional repression, whereas histone acetyltransferase (HAT)-mediated acetylation promotes open chromatin and gene transcription. Created in BioRender. Mohan, A. (2026). https://BioRender.com/5s8xuvt, accessed on 1 August 2026.

Furthermore, preclinical studies have demonstrated that the pharmacological inhibition of HDACs restores histone acetylation, enhances neuroplasticity-related gene expression, and produces antidepressant-like behavioral effects [17]. The main disadvantage of current HDAC inhibitors is their lack of specificity, as they act on several forms of deacetylases. This is the main reason that limits their clinical applicability due to significant side effects [18]. To formulate treatment options that are less toxic and more efficient, research is increasingly focused on selective HDAC inhibitors [19]. Targeting HDAC complexes and isoform-specific inhibitors is gaining popularity in the development of HDACis [20]. The synthesis of pan-HDAC inhibitors with strong activity has already been achieved; thus, specific HDAC inhibitors are now at the center of HDAC drug discovery efforts. Some structural moieties can be incorporated into HDAC inhibitors for isoform specificity [21] and can help us identify the right strategy in drug development [22]. Depression is also associated with other isoforms of HDAC. Different forms of HDAC have shown regional specificity for expression in both cells and brain regions, and changes in expression for different forms are also dependent on tissue type and the stage of depression. There are reports of changes in HDAC2 and HDAC5 expression depending on context in peripheral leukocytes, the hippocampus, and the nucleus accumbens. Imipramine treatment has also shown changes in HDAC5 expression in socially defeated mice [23]. The functions of HDAC2/3 are stage-specific, while HDAC4/5, HDAC1, HDAC9, and SIRT2 affect stress vulnerability, neuroinflammation, or antidepressant sensitivity via region-specific effects [16,24,25,26,27]. The classification, principal functions, and disease involvement of individual HDAC isoforms are summarized in Table 1. HDAC3 and HDAC6 provide a particularly complementary therapeutic framework, so therapeutic approaches for treating depression are increasingly exploring HDAC3 and HDAC6 as targets since they are involved in neuroplasticity, stress response, and cytoskeletal dysfunction [9,10]. The classification of HDACs and the opposing actions of HDAC- and HAT-mediated chromatin regulation are illustrated in Figure 1.

### 2.1. HDAC3: Structure, Localization, and Functional Significance in the Brain

**Figure 2:**
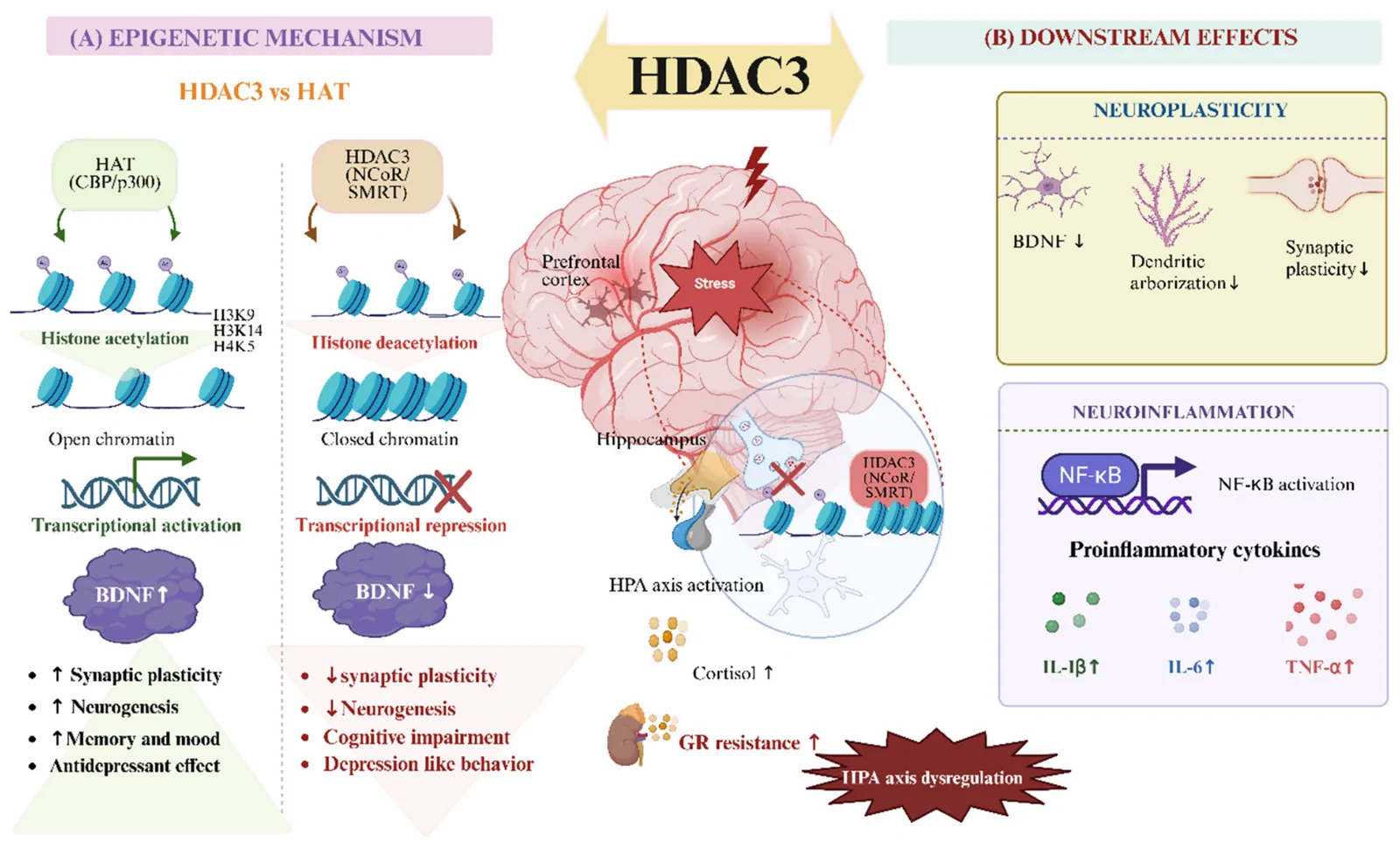
The role of HDAC3 in the pathophysiology of depression. HDAC3-mediated histone deacetylation promotes transcriptional repression. This potentially impairs Synaptic plasticity, enhancing neuroinflammation and hypothalamic–pituitary–adrenal (HPA) axis dysregulation and Depression-like behavior. Conversely, HAT-mediated histone acetylation enhances gene expression and may exert antidepressant effects. Upward (↑) and downward (↓) arrows indicate increases and decreases. Green represents the beneficial effects whereas Red represents the detrimental effects associated with HDAC3 mediated acetylation and deacetylation. Created in BioRender. Mohan, A. (2026) https://BioRender.com/5rpds7h, accessed on 1 August 2026.

Histone deacetylase 3 (HDAC3) is the most highly expressed class I HDAC in the brain and is abundantly expressed in the cortex, hippocampus, and cerebellum. It is primarily expressed in neurons and also present in oligodendrocytes. The enzyme is mostly found in the nucleus, although it is also found in the cytoplasm and plasma membrane. As shown in Figure 2, HDAC3-NCoR/SMRT transcriptional repression occurs through enhanced histone affinity to the negatively charged DNA backbone, causing a condensed chromatin state that blocks transcription, and through the decreased association of bromodomain coactivators, such as chromatin-associated proteins and HATs, that typically bind acetyl-lysine residues and promote transcription. Therefore, unliganded nuclear receptor inhibition involves the HDAC3-NCoR/SMRT pathway [68]. Class I HDAC3 has the typical HDAC domain architecture with an active tunnel-like site and a tyrosine residue near it that influences substrate specificity. HDAC3 targets lysine residues on histones such as H3K9, H3K14, and H4K5. The inactivation of HDAC3 results in increased acetylation and gene expression [69].

In progenitor cells and neurons, HDAC3 is an important isoform that acts as a synaptic plasticity-related gene repressor. HDAC3 gene inactivation results in aberrant behaviors such as hyperactivity and anxiety, as well as post-weaning death due to developmental abnormalities in the neocortex, hippocampus, and corpus callosum [70].

Immunofluorescence microscopy using a particular anti-HDAC3 antibody shows that HDAC3 is widely expressed in the key neurogenic regions of the developing brain, including the ventricular zone (VZ), subventricular zone (SVZ), cortical plate, and hippocampus [70,71,72], highlighting its crucial function in neuronal development and differentiation. Functional investigations indicate that removing HDAC3 in the dorsal hippocampus increases the expression of activity-dependent genes such as Nr4a2 and c-Fos, enhances histone acetylation at H4K8, and promotes long-term memory formation following subthreshold training [72]. Furthermore, BDNF and Npas4, essential regulators of synaptic plasticity and neuronal viability, are transcriptional targets of HDAC3 suppression. The pharmacological inhibition of HDAC3 mitigates neuronal loss and behavioral impairments in neurodegenerative models [73]. HDAC3 controls cell survival, inflammation, and metabolism. Its overexpression produces neuron-specific toxicity via the Akt–GSK3β pathway [74].

In contrast, its deficiency in microglia and macrophages modulates inflammatory responses. It enhances neuroprotective effects by increasing histone acetylation, derepressing inflammatory genes, decreasing nitric oxide generation, and promoting phagocytic activity [75,76]. In addition to its brain actions, HDAC3 plays a role in tissue remodeling and metabolic homeostasis; its deletion influences atherosclerotic plaque stability and hepatic metabolism through PPARγ and mTOR signaling [77,78]. At the genomic level, HDAC3 is crucial for preserving chromatin architecture and genomic integrity, with its dysregulation associated with oncogenic signaling pathways, such as prostate cancer, where pharmacological suppression diminishes tumor development and proliferation [79,80,81].

### 2.2. Role of HDAC3 in Neuroinflammation, Synaptic Plasticity, and Depressive Phenotypes

Histone deacetylase 3 (HDAC3) has been identified as a significant epigenetic regulator implicated in brain function and neuroinflammatory mechanisms, synaptic plasticity, and stress-related behavior. Growing evidence shows that the dysregulation of HDAC3 has a role in the onset and advancement of major depressive disorder (MDD) [37,73,82]. HDAC3 inhibition induces antidepressant properties through a reduction in proinflammatory cytokines, improvement in neuronal functions, and stimulation of neuroplasticity within specific brain regions. This information establishes that HDAC3 is a potential therapeutic target in the treatment of mood disorders [37,83]; however, evidence for the direct antidepressant activity remains largely preclinical.

For example, in an LPS (Lipopolysaccharide)-induced depression model, RGFP966 reduced proinflammatory cytokine production and microglial activation and depressive-like behaviors in mice [37]. The inhibition of HDAC3 has also been shown to affect neuroimmune responses in depression. In an immune-challenge rat model, inhibiting HDAC3 reduced depressive-like behavior and repaired aberrant microglial morphology in the amygdala in a sex-dependent manner [84]. Studies have shown that HDAC3 is involved in both neuroprotective and neuroinflammatory functions in the CNS. The inhibition of HDAC3 reduces the neuroinflammatory response and promotes neuroregeneration and synaptic plasticity; the increased expression of HDAC3 is associated with an enhanced inflammatory response in microglia. It is suggested that HDAC3 represses the expression of inflammation resolution genes through histone deacetylation, leading to neuroinflammation, and on the other hand, through its interaction with non-histone proteins, it regulates transcription factors and signal transduction pathways responsible for neuroprotection. In this way, the function of HDAC3 depends on cell type and condition, which may account for the fact that HDAC3 inhibition is advantageous in active pathology but not under the proper conditions of HDAC3 function [83]—supporting the relevance of progressive neuroinflammation to stress susceptibility. In a study by BRAVO-Tobar et al., the social defeat stress gradually induced hippocampal neuroinflammation along with behavioral signs of depression in rats. Although some subjects seemed to display initial resilience to stress-induced behaviors, they ultimately lost that resilience as neuroinflammatory alterations progressed and correlated with the onset of behavioral depression. In conclusion, stress-induced resilience was transient and was marked by increasing levels of inflammation in the hippocampus [85]. Collectively, while the anti-inflammatory activity of HDAC3 inhibition is relatively consistent in experimental settings, the impact of HDAC3 inhibition on depressive behavior, learning, and memory has been demonstrated in multiple models and is thus not mechanistically equivalent.

The effects of HDAC3 are context-dependent. In chronic stress models, class I HDAC inhibition in the prefrontal cortex, which includes HDAC3 among others, has antidepressant-like effects [86]. HDAC3 plays an important role in the glucocorticoid (GC)-induced tethered transrepression of inflammatory genes, a relevant mechanism in major depressive disorder (MDD). Although inhibiting HDAC3 has demonstrated antidepressant-like effects in various experimental models, the proper nuclear localization of HDAC3 may also signify a protective adaptive response to persistent stress. In a chronic social defeat stress model, Athira K. V. et al. showed that resilient mice had higher amounts of the HDAC3 protein in their nuclei than control and stress-susceptible mice. The simultaneous rise in nuclear HDAC3 and glucocorticoid receptor (GR) levels indicates the heightened inhibition of inflammatory gene transcription, hence promoting stress resilience.

On the other hand, lower levels of nuclear HDAC3 and GR signaling may make it harder to control inflammation and increase susceptibility to depressive-like behaviors [87]. These apparently conflicting findings indicate that HDAC3 is not always harmful. The overexpression of HDAC3 activity can impair plasticity, but the nuclear expression of HDAC3 related to glucocorticoid receptor signaling may serve as an adaptive anti-inflammatory response to stress conditions. Hence, the effect can differ according to the location within the brain, cellular context, sex, subcellular localization, and stages of depression.

In hippocampal-dependent object recognition, HDAC3 plays a crucial role in regulating improved memory. Additionally, it was demonstrated that the pimelic diphenylamide drug, RGFP136, may be used as a treatment for cognitive disorders. The same results were observed using both RGFP136 treatment and HDAC3 inhibition (using HDAC3flox/flox and DADm mice). The function of HDAC3 in memory might depend on the interactions between HDAC3 and NCoR [72]. The targeted inhibition of HDAC3 activity in the dorsal hippocampus or basal nucleus of the amygdala augmented contextual fear memory. In contrast, suppression in the lateral nucleus specifically improved tone-associated fear memory, demonstrating that HDAC3 modulates distinct facets of associative memory across various amygdala subregions [88].

RGFP966 similarly enhanced auditory associative memory and developed persistent cortical plasticity, implying that HDAC3 negatively impacts memory fidelity and experience-based neuroplasticity [82]. A study using PT3, a novel selective HDAC3 inhibitor, showed in a unique object recognition mouse model that it enhanced blood–brain barrier permeability and long-term memory. PT3 administration enhanced histone H3K9 acetylation in the hippocampus and increased the expression of synaptic plasticity-related genes such as BDNF, CREB1, and PSD95, indicating that selective HDAC3 inhibition improves memory formation via epigenetic regulation of neuroplasticity [89]. This study establishes that HDAC3 is involved in memory-related plasticity, but better cognitive function does not automatically mean an antidepressant effect. In addition, increased fear-related memory formation could even be considered detrimental in stress disorders.

Given the involvement of HDAC3 in neuroinflammatory and stress-related pathways, numerous chemical scaffolds, such as hydroxamates, benzamides, modified benzamides, and hydrazides, have demonstrated inhibitory activity and selectivity against HDAC3 with varying degrees of selectivity over other HDAC isoforms [3]. Consequently, significant research efforts have been directed toward identifying and developing selective HDAC3 inhibitors using computational modeling, structure-based drug design, and pharmacophore-based approaches. The critical structural requirements for HDAC3 inhibition have been determined using the pharmacophoric model and 3D QSAR analysis of benzamide analogs by Goverdhan Lanka et al. Through docking, virtual screening, MM/GBSA binding energy calculations, molecular dynamic simulations, and ADMET predictions, optimized lead compounds with increased stability and binding affinity against HDAC3 were discovered [90]. HDAC3 has been observed to have non-catalytic properties because even catalytically dead mutants can partly compensate for physiological problems. This means that HDAC3 functions not only catalytically but also structurally and/or in regulatory ways, which is important in drug design [91]. Therefore, the inhibition of HDAC3 catalytic activity may not reproduce all the effects of genetic HDAC3 depletion, which remains an important mechanistic uncertainty.

In addition to this functional complexity, the safety properties of HDAC3-selective inhibitors deserve special consideration. Research has demonstrated that HDAC3 knockout induces significant cardiac hypertrophy. Therefore, the evaluation of target specificity at the tissue or cellular level is critical during the development of HDAC3-selective inhibitors [92]. Furthermore, the design of specific HDAC3 inhibitors has been difficult. This is mainly due to the remarkable similarities that exist between all the zinc-dependent isoforms of HDACs. As a result, there are few specific HDAC3 inhibitors [93]. The mechanisms linking HDAC3 activity to major depressive disorder (MDD) pathophysiology are summarized in Figure 2.

### 2.3. HDAC6: Cytoplasmic Deacetylase Regulating Cytoskeletal and Stress-Related Pathways

Histone deacetylase 6 (HDAC6) represents another important isoform with distinct roles in neuronal function and stress responses. Recent studies have focused on non-histone targets of class II HDACs, with particular emphasis on HDAC6 [94]. HDAC6 is a unique member of the HDAC family, specifically involved in cytoplasmic non-histone protein acetylation, and has been implicated in the stabilization of the cytoskeleton, cell motility, and intracellular trafficking. HDAC6 is expressed in brain areas that are associated with memory function, namely the hippocampus and cortex [95].

**Figure 3:**
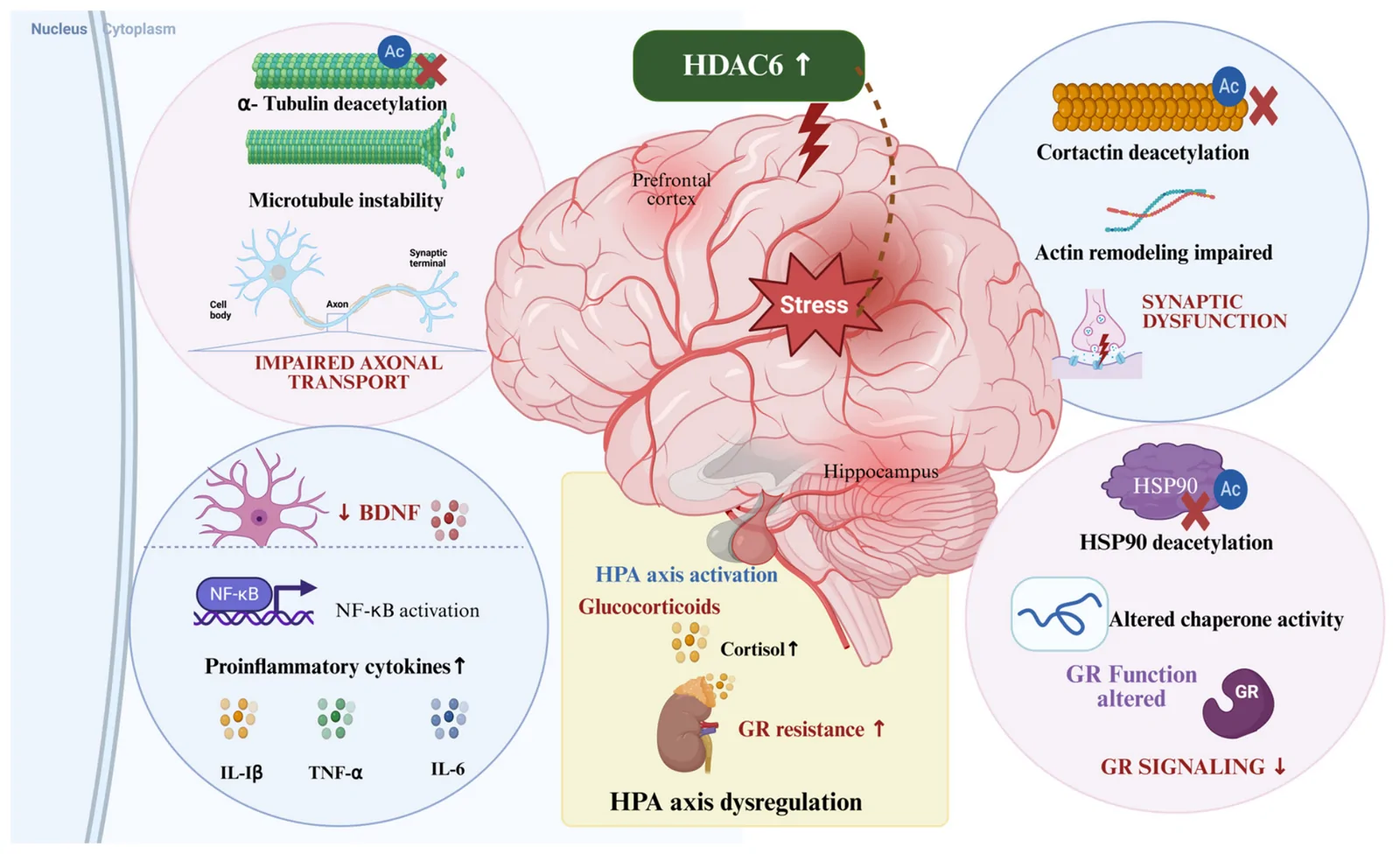
The role of HDAC6 in the pathophysiology of depression. HDAC6 deacetylates non-histone substrates including α-tubulin, cortactin, and heat shock protein 90 (HSP90). Increased HDAC6 activity may impair axonal transport and synaptic function while promoting neuroinflammation and altering GR signaling, leading to HPA axis dysregulation and depressive-like behavior. Upward (↑) and downward (↓) arrows indicate increases and decreases, respectively; crosses indicate deacetylation; dashed lines indicate pathway associations; and coloured panels distinguish the different downstream pathways. Created in BioRender at https://BioRender.com. Mohan, A. (2026). https://BioRender.com/gufpx9h, accessed on 1 August 2026.

HDAC6 is the largest member of the HDAC family, consisting of 1215 amino acids, and uniquely owns two catalytic domains (CD1 and CD2) along with a ubiquitin-binding domain. Despite both domains possessing conserved catalytic residues, the role of CD1 is ambiguous, whereas CD2 predominantly enables the deacetylation of cytosolic substrates and serves as the principal catalytically active domain [96]. HDAC6 is prominently expressed in the brain, where it is recognized for regulating emotional behaviors in rodents. Mice lacking HDAC6 exhibit hyperactivity, reduced anxiety, and a diminished depressive phenotype, suggesting that the acetylation state regulates the cellular mechanisms involved in emotional modulation [97]. The best-known substrate of HDAC6, is *α*-tubulin, and other substrates have been identified, including Hsp90 and cortactin [98]. α-Tubulin is acetylated and deacetylated at the lysine 40 (K40) position via acetyltransferase and deacetylase enzymes, respectively. Increased tubulin acetylation resulted in behavioral effects that resembled those caused by traditional antidepressants. The proposed roles of HDAC6 in depression are summarized in Figure 3. HDAC6, a cytosolic histone deacetylase, is recognized for its ability to deacetylate α-tubulin. Altered tubulin post-translational modifications in major depressive disorder (MDD) are associated with disrupted cytoskeletal organization and synaptic dysfunction. Proteomic and imaging studies also demonstrate stress-induced dendritic retraction and regional brain atrophy in the hippocampus, prefrontal cortex, and amygdala. These findings indicate that microtubule dysfunction may be a common feature of neuropsychiatric disorders [97]. HDAC6 inhibition has been shown to produce antidepressant-like effects in rodent models of depression [99].

HDAC6 binds to cortactin and controls its acetylation levels [100]. Cortactin associates with F-actin and facilitates the assembly of actin filaments [101]. HDAC6 mediates the deacetylation of lysine residues present within the repeat domain of cortactin, enabling the association of Arp2/3 complex proteins to promote actin branching [102]. Branching F-actin is involved in forming a structural network that serves as a scaffold in spine formation and maintenance in dendrites. The Arp2/3 complex participates in spine formation and synapse plasticity [103]. Cortactin is a critical component of synaptic plasticity because of its influence on actin cytoskeletal remodeling and synaptic morphology. In addition to its involvement at the postsynaptic level, cortactin is involved at the presynaptic level by participating in neurotransmitter release through the regulation of the active zone and synaptic vesicles [102].

One of the important molecular chaperones targeted by lysine deacetylases [104], heat shock protein (HSP90), is modulated through deacetylation via HDAC6, which increases its chaperone function and stabilizes client proteins like the glucocorticoid receptor (GR) [105,106]. HSP90 and HDAC6 interact and play a role in activating HSF1 and heat shock protein genes [105]. The GR-HSP90 complex, along with its co-chaperones FKBP51/52, plays a major role in signaling and nuclear localization in the presence of stress hormones. HDAC6 inhibition impairs function and reduces responses to stress and glucocorticoids. The impaired negative feedback regulation of the hypothalamic–pituitary–adrenal (HPA) axis is a central feature of the glucocorticoid receptor hypothesis of depression, resulting in excessive cortisol release and structural changes in stress-sensitive brain regions. This process is regulated by the HDAC6-mediated acetylation of HSP90, affecting GR development, nuclear transport, and stress-induced transcriptional responses [107]. In addition, the inhibition and knockdown of HDAC6 and its target HSP90 hinder the elevation of glutamate transmission induced by stress in the prefrontal cortex [108]. Acute and chronic stress actions involve interconnected and related mechanisms, such as the need for GR signaling. Unlike acute stress, which is adaptive, chronic stress can induce maladaptive changes in neuronal activity by causing dendritic retraction, blocking synaptic plasticity, and inhibiting neurogenesis. The inhibition of HDAC6 might therefore be able to prevent the detrimental changes that are seen during chronic stress and provide neuroprotective action against stress-induced mental illness [109]. Although the functions of α-tubulin, cortactin, and HSP90 are critical for development, HDAC6-deficient mice are viable with normal development and do not exhibit noticeable defects in brain morphology, hippocampal-dependent cognitive functions, or motor coordination [102]. HDAC6 also plays a critical role in the autophagy–lysosome pathway, maintaining protein quality control during aberrant protein aggregation [110]. In addition, HDAC6 is important in neural cell development by facilitating neurite growth via the activation of the centrosomal Cdc20-APC complex [101]. The selective inhibition of HDAC6 leads to neuroprotection against oxidative stress and microtubule stabilization [111]. HDAC6 also supports cell survival by mediating aggresomal formation and the transport of misfolded proteins [112].

In addition to its role in stress biology and synaptic plasticity, HDAC6 has been implicated in other pathological conditions, including neurodegenerative diseases and cancer, where it regulates tau phosphorylation, inflammatory signaling, and tumor cell migration [105,113,114,115].

### 2.4. Involvement of HDAC6 in Stress Response, Neuroplasticity, and Depression

Histone deacetylase 6 (HDAC6) is a potential therapeutic target for the CNS [116]. Recently, different studies have identified the role of HDAC6 in models of stress adaptation and shown that HDAC6 functional deficiency results in an effect similar to that of an antidepressant phenotype [98]. Through its cytoplasmic substrates, HDAC6 links cytoskeletal and chaperone regulation to stress adaptation and depressive-like behavior [117].

Selective HDAC6 inhibitors have shown relatively consistent antidepressant effects in preclinical models. Tubastatin A reduces anhedonia and behavioral despair within 24 h in corticosterone-treated rats. The stabilization of microtubules, acetylation of α-tubulin, ERK pathway activation, and modulation of dopaminergic and serotonergic neurotransmission were associated with these effects [118]^.^ Similarly, the brain-penetrant inhibitors ACY-738 and ACY-775 enhance α-tubulin acetylation without altering histone acetylation, leading to high brain bioavailability and antidepressant effects in animal trials. These inhibitors indicate that HDAC6 could be a novel target for depression therapy. It increases exploratory behavior and potentiates the effects of selective serotonin reuptake inhibitors (SSRIs), such as citalopram [98].

Nevertheless, the relationship between HDAC6 expression and stress-related behavior appears to be context-dependent. A study on agomelatine, a chronobiological antidepressant drug, was conducted to determine whether agomelatine could reverse stress-induced behavioral changes and alter hippocampal gene expression in response to stress. Chronic social defeat stress (CSDS) leads to behavioral impairments in C57BL/6J mice. RT-qPCR analysis showed a significant reduction in HDAC6 mRNA levels in the mouse hippocampus subjected to CSDS, which was counteracted by the chronic administration of agomelatine. Since there were no measurements of HDAC6 protein expression levels and its enzymatic activity, this observation cannot support reduced HDAC6 activity and therefore does not contradict the antidepressant-like effects of HDAC6 inhibitors. In addition, the social stress-induced inhibition of MAP-2, along with BDNF exon IV, can lead to dendritic or synaptic changes that could cause cognitive dysfunction. In contrast, chronic agomelatine therapy might prevent deficits in BDNF exon IV and MAP-2 expression [119]. The restoration of reduced HDAC6 expression by agomelatine appears to differ from studies reporting the beneficial effects of HDAC6 inhibition.

Another study showed that the HDAC6-dependent regulation of GR chaperone dynamics in the dorsal raphe nucleus (DRN) is a key mechanism underlying resilience to psychosocial stress. Chronic social defeat stress (CSDS) increases nuclear GR levels in vulnerable mice, which is associated with the decreased acetylation of Hsp90. Both genetic and pharmacological enhancement in Hsp90 acetylation reduce GR signaling in serotonergic neurons and protect against the development of social avoidance in the CSDS model [48,107]. These results suggest a mechanistic role in which the deacetylation of HSP90 by HDAC6 enhances GR function, whereas the acetylation of HSP90 inhibits GR activation, thereby providing a neuroprotective response against stress. However, this mechanism was demonstrated specifically in the serotonergic neurons of the DRN and should not be generalized to all brain regions or cell types.

Another HDAC inhibitor, vorinostat (VOR), produces antidepressant-like effects in chronic social defeat stress (CSDS) mice through the modulation of cytoplasmic HDAC6, indicating that resilience and susceptibility to CSDS are associated with differential nuclear levels of HDAC6 in the hippocampus, indicating their role in stress-adaptive versus maladaptive responses [87]. Because vorinostat inhibits different HDAC isoforms, these findings cannot establish that its antidepressant-like effects are mediated specifically by HDAC6. Under CSDS conditions in C57BL/6 mice, both resistant and susceptible phenotypes were generated based on social interaction behavior after experiencing the stress. Susceptible mice demonstrated anhedonia and anxious behaviors, along with deficits in learning and memory. Changes occurred as a result of decreased levels of postsynaptic density protein PSD95, PSD93, PKA, and phosphorylated CREB (cAMP response element-binding protein) in the hippocampus, indicating that synaptic signaling pathways and cAMP response element-binding protein signaling are disrupted. High levels of HDAC6 were also identified in the susceptible phenotype, indicating its activity in stress-induced synaptic dysfunction and cognitive impairment [120].

Based on their distinct cellular functions and localizations, HDAC3 and HDAC6 play different roles in depression.

## 3. Sex Differences in Stress-Related HDAC Biology

Biological sex is another contributor to stress response variations since women have more vulnerability to stress-induced illnesses than men. In this respect, there is clinical and preclinical evidence for sex-dependent differences in endocrine, emotional, arousal, and inflammatory stress responses. Such differences may stem from sex-dependent differences in how an individual experiences stress, sex-dependent differences in stress responses and limbic pathways, and interactions among gonadal hormone function, corticotropin-releasing factor (CRF) signaling, the activity of the hypothalamic–pituitary–adrenal (HPA) axis, glucocorticoid receptor sensitivity, and neuroimmunity. Therefore, the same stressor can lead to different molecular, neuroendocrine, and behavioral adaptations in males and females, resulting in sex-specific vulnerability and resilience to stress. However, the exact mechanisms underlying such differences are not fully elucidated. Therefore, studies need to involve both sexes and analyze sex-by-stress interactions [121,122,123,124].

Historically, many studies in preclinical neuroscience and stress fields have used mainly male animals or have not performed a separate analysis based on sex [121,125,126,127]. This leads to insufficient representation and, therefore, an inability to conclude about possible differences in efficacy, pharmacokinetics, pharmacodynamics, or adverse effects between males and females [128,129]. Therefore, future HDAC3 and HDAC6 studies should use two sexes and report outcomes based on the sex of participants [121,125,127]. Information about estrous cycle stage can be collected or analyzed when relevant to the study design; however, possible hormonal variations should not be used as a reason to exclude female animals from further study [126,127]. These studies are needed to establish whether sex-specific approaches to dosage, administration time, or patient stratification are needed for treatments targeting HDAC3 and HDAC6.

In addition to these methodological and translational considerations, the sex-dependent regulation of histone acetylation and BDNF-mediated synaptic plasticity was found in animal models of trauma and early life stress, suggesting that stress-linked epigenetic regulation may differ in male and female animals [128,129]. Yet, the data regarding the role of HDAC3 and HDAC6 specifically in this context are limited; thus, this remains a novel hypothesis.

## 4. Comparative Mechanisms of HDAC3 and HDAC6 in Depression Pathophysiology

Histone deacetylase 3 (HDAC3) and histone deacetylase 6 (HDAC6) differ fundamentally in their subcellular localization and functional roles in neuronal regulation [68,95]. Mechanistically, these two deacetylases contribute to neuronal activity modulation through distinct yet complementary pathways. The acetylation and deacetylation of histones constitute fundamental epigenetic mechanisms crucial for regulating transcription and gene expression. Specifically, the deacetylase activity of HDAC3 depends on its interaction with the deacetylase-activating domain of particular nuclear corepressors, namely N-CoR (nuclear receptor corepressor) and SMRT (silencing mediator for retinoid and thyroid receptors). As the complex’s catalytic element, HDAC3 connects transcriptional repression with histone deacetylation. HDAC3 can deacetylate non-histone transcriptional regulators. This could change how transcription is controlled. For instance, HDAC3 can deacetylate both P300 and CBP, which stops them from doing their HAT tasks. N-CoR and HDAC3 both interact directly with CBP. Additionally, HDAC3 can deacetylate NF-κB component RelA, which facilitates its nuclear export and stops NF-κB signaling [83]. Histone deacetylase 3 (HDAC3) has been reported to efficiently inhibit NFκB activation, which is further known to regulate synaptic plasticity [130]. The constitutive NFκB identified in several brain regions, such as the cortex, hippocampus, and cerebellum, is found within the nucleus and is transcriptionally active. Importantly, NF-κB signaling is calcium-dependent and regulated by synaptic activity. It is activated from its cytoplasmic IκB-bound form by stimuli such as glutamate, kainate, TNF-α, oxidative stress, and depolarization. Once activated, NF-κB regulates PKA and CREB signaling, forming a transcriptional cascade involved in synaptic plasticity and memory [83,131]. Dysregulated calcium homeostasis in neurons plays an important role in the pathogenesis of depression by causing various pathological changes, such as synaptic loss, impaired neurogenesis, neuroinflammation, and the dysfunction of the HPA axis [132]. Consistent with its role in plasticity, HDAC3 negatively regulates memory by repressing plasticity-related genes such as BDNF and Npas4. Accordingly, the inhibition of HDAC3 increases spine density and enhances synaptic markers, including synaptophysin, BDNF, and p-CREB in the hippocampus [83,133].

In contrast, HDAC6 primarily regulates cytoplasmic processes related to cytoskeletal dynamics and protein trafficking. HDAC6 inhibition increases acetylated α-tubulin levels, promoting BDNF trafficking and activity-dependent secretion [134]. In hippocampal neurons, AKT knockdown negatively affects synaptogenesis and reduces dendritic spine density. In contrast, increased AKT acetylation is observed in human neural cells treated with HDAC6 inhibitors, particularly at the kinase domain residues K163 and K377. Thus, by altering AKT acetylation at K163 and K377, HDAC6 may regulate synaptic function and plasticity [135].

Apart from its role in regulating synaptic function, HDAC6 participates in inflammatory signal transduction pathways, where it has been identified as a potential drug target because of its unique presence [136]. The enzyme alters signaling pathways crucial for inflammatory processes, such as NF-κB and MAPK. HDAC6 mediates the deacetylation of the p65 subunit of NF-κB, thus allowing the protein complex to translocate into the cell nucleus and initiate transcription, leading to higher gene expression associated with inflammation and immunity [137].

Furthermore, glucocorticoid receptor (GR)-mediated transcriptional repression is controlled by epigenetic mechanisms involving histone deacetylases. HDAC3 plays a crucial role in GR-dependent transrepression [138]. However, HDAC6 also regulates GR signaling. Specifically, when HDAC6 is depleted, Hsp90 becomes hyperacetylated. This change affects the cellular response to glucocorticoid hormones [48].

**Figure 4:**
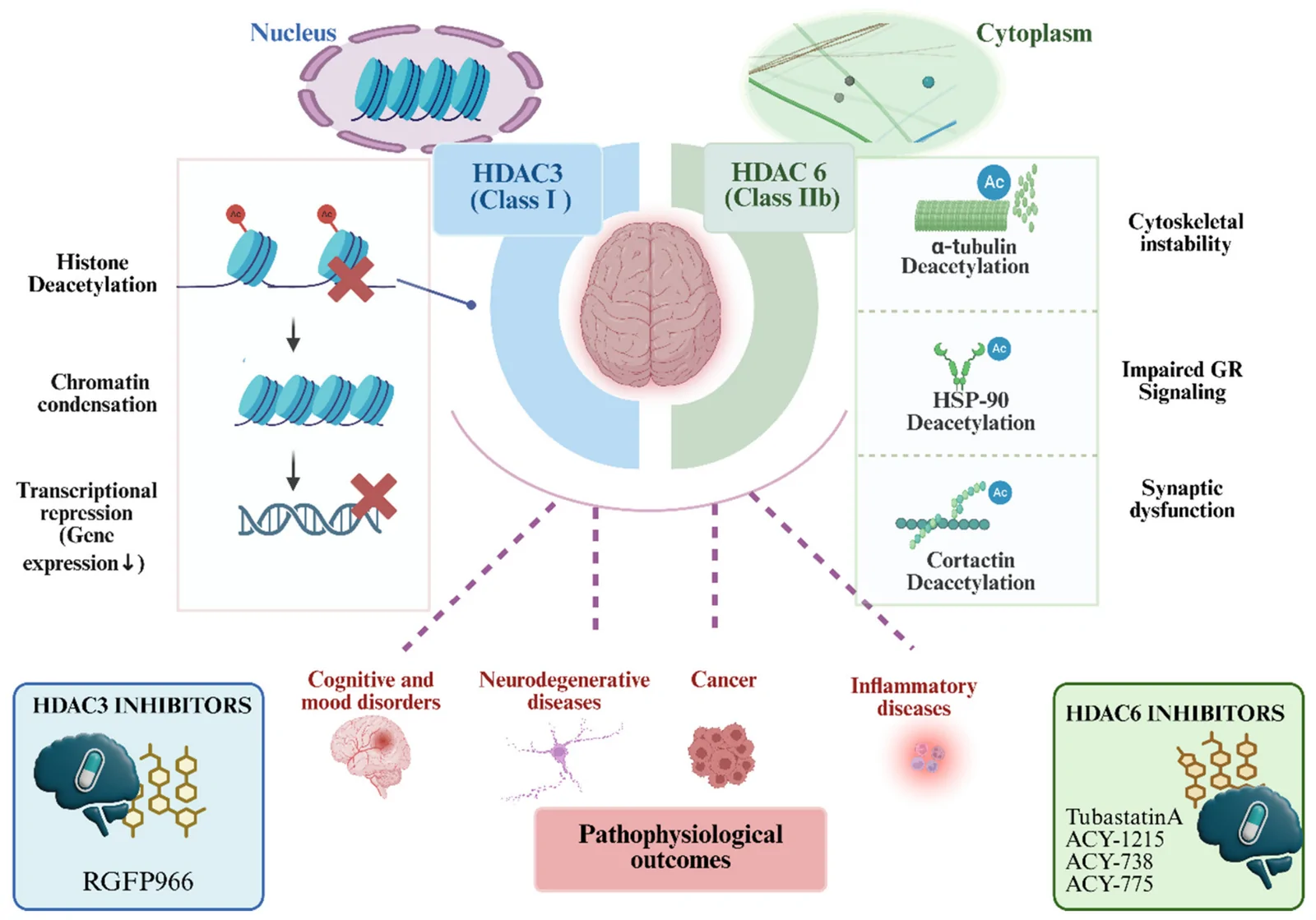
Comparative roles of HDAC3 and HDAC6 in neurological disorders. HDAC3 primarily mediates nuclear epigenetic regulation, whereas HDAC6 regulates cytoplasmic signaling and cytoskeletal dynamics. Dysregulation of either enzyme may contribute to neuroinflammation and neurological disorders. Blue and green represent HDAC3 and HDAC6 pathways, respectively; arrows show progression, dashed lines show associations, red crosses show inhibition. Created in BioRender. Mohan, A. (2026). https://BioRender.com/f4cx5px, accessed on 1 August 2026.

Overall, HDAC3 and HDAC6 are crucial mediators of GR signaling [87], acting at distinct but converging levels of neuronal regulation, where HDAC3 controls transcriptional programs underlying synaptic plasticity. At the same time, HDAC6 regulates cytoskeletal dynamics, protein trafficking, and stress-responsive signaling, as compared in Figure 4. Therefore, dual inhibition may produce synergistic effects by simultaneously enhancing gene expression-dependent synaptic remodeling and restoring the cytoskeletal and signaling pathways required for neuronal connectivity and stress adaptation.

## 5. Scientific Rationale for Dual HDAC3/HDAC6 Targeting

Histone deacetylase 3 (HDAC3) plays an important role in many stages of neurodevelopment, as shown by molecular genetic studies and isoform-specific inhibitors. It is an important regulator of synaptic plasticity and memory development, with beneficial and detrimental functions. Although HDAC3 is essential for normal brain development, in the adult brain it contributes to neurodegenerative and neuropsychiatric processes, suggesting a context-dependent function [139].

In the mature brain, HDAC3 inhibits memory development. Both the administration of RGFP966 and the hippocampal-specific deletion of HDAC3 in adult animals have demonstrated this [72,140]. The effect is mediated through the transcriptional regulation of the immediate early genes (IEGs) c-Fos and Nr4a2, which are both known to be involved in memory formation and enhancing synaptic plasticity [141,142,143,144]. The expression of c-Fos and Nr4a2 genes is increased when HDAC3 is deleted because it binds to their promoters [140].

In contrast to the transcriptional role of HDAC3, histone deacetylase-6 (HDAC6) primarily regulates synaptic function through cytoplasmic mechanisms [48]. HDAC6 inhibition facilitates α-tubulin acetylation, which in turn allows for the transport of molecules like brain-derived neurotrophic factor (BDNF), thus enhancing neuroplasticity [145]. In the prefrontal cortex, HDAC6 inhibition or knockdown protects against acute stress-induced increases in glutamatergic transmission and abnormal glutamate receptor trafficking, thereby preserving synaptic plasticity [109].

Together, all evidence indicates that HDAC3 regulates the transcriptional program necessary for the formation and maintenance of synaptic plasticity, whereas HDAC6 manages the structural and functional machinery required to carry out and stabilize synaptic modifications. Thus, HDAC6 is a promising treatment target for major depressive disorder [145]. A dual HDAC3/HDAC6 inhibitor, compound 24c, exhibited potent inhibitory activity (IC50 values of 2.6 and 34 nM) in enzymatic assays, with low nanomolar efficacy against both HDAC3 and HDAC6 and marked selectivity (approximately 300–3000-fold) relative to other HDAC isoforms, supporting the feasibility of simultaneous targeting. It was weaker against HDAC1/2 (~1 µM) and showed no detectable inhibition of HDAC4/5/7/9 at 10 µM, supporting its selective dual HDAC3/HDAC6 profile. Immunoblot analysis in KMS-12-BM cells showed that 24c enhanced the acetylation of histones H3 and α-tubulin, validating the inhibition of both HDAC3 and HDAC6 and supporting the in vitro findings. However, both 24c and the broad-spectrum HDAC inhibitor 3 increased H3K9Me2 modification, implying that HDAC inhibition alone is insufficient and requires the simultaneous use of H3K9-specific methyltransferase inhibitors. Based on the in vitro ADME and toxicity studies, compound 24c has a high permeability profile, good metabolic stability, low toxicity to normal hepatocytes and cardiomyocytes, and no genotoxic activity, making it a suitable candidate for further in vivo studies [11]. The HDAC3 and HDAC6 inhibitory activities, selectivity profiles, and reported pharmacological effects of compound 24c and other representative HDAC inhibitors are summarized in Table 2. Compared with isoform-selective or pan-HDAC inhibitors, this dual approach might result in synergistic outcomes owing to the different functions of HDAC3 and HDAC6; however, the mechanisms underlying this possibility still need to be thoroughly investigated in depression studies because its BBB penetration, CNS exposure, antidepressant efficacy, and long-term safety remain unknown.

**Figure 5:**
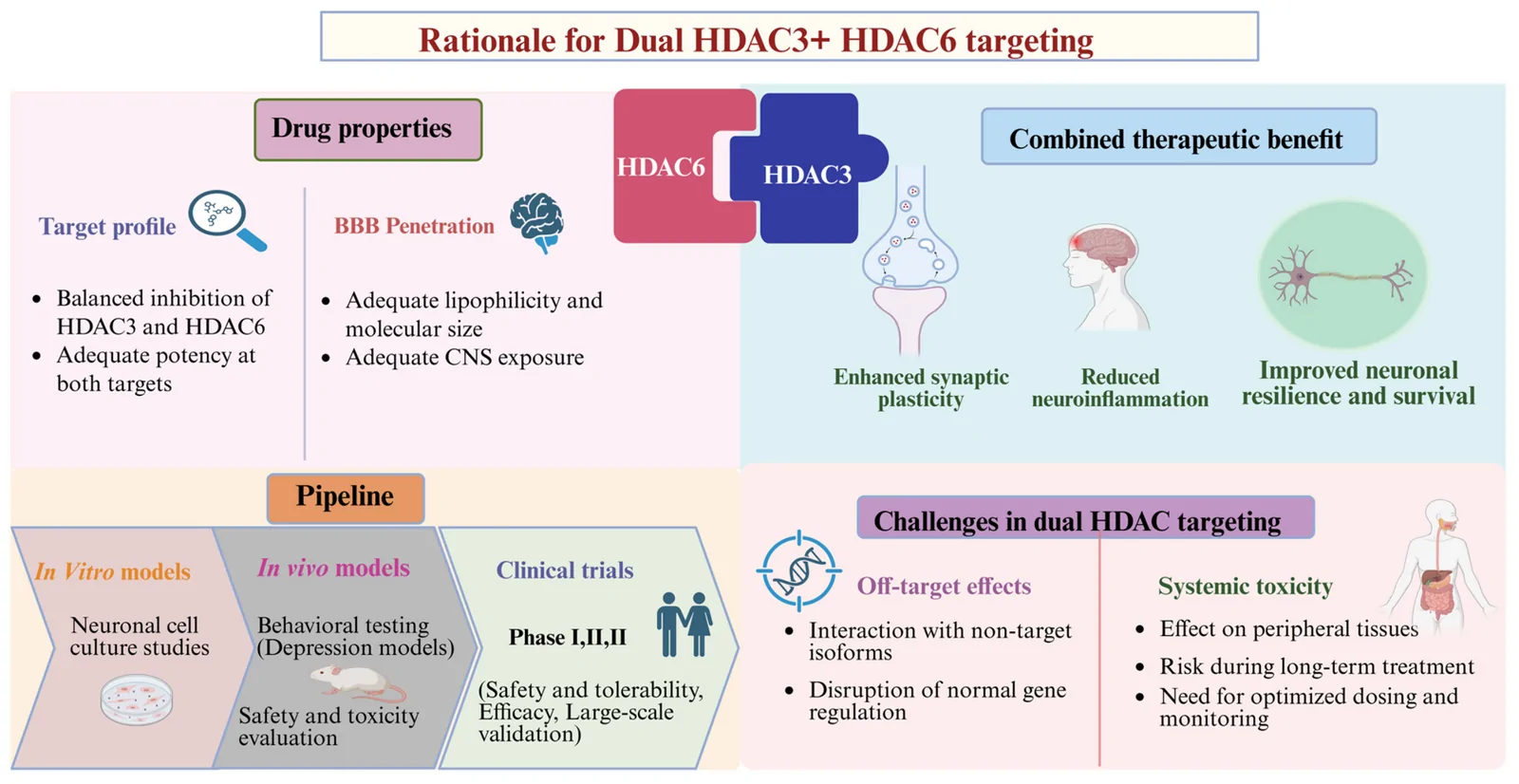
Therapeutic rationale for dual HDAC3/HDAC6 inhibition in depression. Concurrent inhibition of HDAC3 and HDAC6 may improve neuroplasticity and reduce neuroinflammation by targeting complementary nuclear and cytoplasmic pathways. However, challenges related to safety, toxicity, and selectivity must be addressed. Created in BioRender. Mohan, A. (2026). https://BioRender.com/69dyi2h, accessed on 1 August 2026.

As shown in Figure 5, targeting HDAC3 and HDAC6 simultaneously may result in a more comprehensive and successful restoration than targeting either one alone. Because depression involves several interrelated impairments in neuroplasticity, gene regulation, and synapse function, focusing simply on one pathway may result in inadequate repair.

## 6. Translational Challenges in HDAC3/HDAC6-Based Antidepressant Development

As depression is associated with complicated pathophysiological mechanisms, and the available monoaminergic drugs have limited effectiveness, it continues to be a major public health issue. There is evidence supporting an interplay between environmental factors and genetically determined susceptibility through epigenetic changes that regulate gene expression. Chromatin epigenetics has gained ground as a potential mechanism for treating depression [68].

However, despite this promise, limited brain penetration remains a major challenge for histone deacetylase inhibitors (HDACis) in central nervous system (CNS) therapy. Early studies using carbon-11-labeled HDAC inhibitors, such as valproic acid, butyric acid, and 4-phenylbutyric acid, reported minimal blood–brain barrier (BBB) permeability. Similarly, entinostat and vorinostat have shown poor brain uptake or limited behavioral effects due to inadequate BBB penetration [156].

Furthermore, many HDAC inhibitors used for brain disorders fail to cross the BBB effectively, often requiring high doses to achieve therapeutic effects, which may be due to low potency, poor selectivity, or insufficient brain exposure [91]. In addition, most selective HDAC6 inhibitors have poor BBB penetration, which decreases the therapeutic effect on neuroinflammatory disorders and results in low drug concentration in the brain tissue [157].

In neurodegenerative disease animal models, panobinostat proved to be highly efficient in crossing the blood–brain barrier and producing histone hyperacetylation, indicating its neuroprotective effect [155]. Together, these findings emphasize the need for systematic strategies to improve the BBB permeability of HDAC inhibitors for effective CNS treatment. BBB penetration can be improved by optimizing physicochemical properties such as molecular weight, lipophilicity, polar surface area, hydrogen-bonding capacity, and metabolic stability [158]. Alternative approaches, including prodrug development, nanoparticle-based delivery, and intranasal administration, may also improve brain exposure, although their safety and delivery efficiency require further validation [159,160].

The positron emission tomography (PET) tracer [^18F] PB200 is a selective and penetrable compound for use in the brain, which binds with high affinity (nanomolar) to HDAC6 and has very good stability in vivo. Consistent with the findings from rodent and non-human primate models, PET imaging revealed that patients with major depressive disorder have increased levels of HDAC6 that are co-localized with activated microglia and other neuroinflammatory pathways; therefore, there seems to be an association between the upregulation of HDAC6 and neuroinflammation in depression. Overall, the tracer contributes as a valuable tool for assessing HDAC6 target engagement/responses in therapeutic studies of neuropsychiatric disorders [161].

Histone acetylation usually displays rapid turnover, especially during gene transcription [162]. In contrast, the effects of DNA methylation might be more durable and specific to certain genes [163]. The therapeutic effect of HDAC inhibitors depends on their kinetic parameters and drug exposure duration. In cancer cells, the rapid dissociation of hydroxamates allows for the rapid recovery of HDAC function upon drug removal, while slower benzamide binding results in different temporal changes in histone acetylation and gene expression. However, all these data cannot be generalized to neurons and depression, and further study is needed [164].

Apart from pharmacokinetic limitations, pan-selective HDAC inhibitors can target not only histones but also other cellular proteins such as cytoskeletal proteins, transcription factors, and nuclear transport proteins, thereby affecting cell cycle regulation, differentiation, and even apoptosis. Such effects have the potential to cause some toxic reactions in cells [165]. To address these issues, isoform-selective HDAC inhibitors are greatly desired for studying the roles of individual isoforms [166]. Selective inhibitors that target a single HDAC isoform or class subtype are expected to be less toxic and more tolerated. Structural modifications and the inclusion of new chemical moieties may help evolve isoform selectivity [167]. Isoform selectivity could be achieved through the design of the cap and linker region of the inhibitor via a structure-based approach or through the addition of interactions with the specific isoform side, lower, and foot pockets near the catalytic site [168].

Since HDAC inhibitors control a wide range of genes, blocking different isoforms may lead to positive or negative epigenetic outcomes. Thus, specific HDAC inhibitors are considered more favorable than non-specific ones [21]. It has been established that the simultaneous inhibition of HDAC3 and HDAC6 leads to synergistic effects [169], supporting the rationale for targeted multi-inhibition strategies.

Despite these advances, HDAC inhibitors, comprising vorinostat, valproic acid, romidepsin, panobinostat, and entinostat, have been tested in several clinical phases from phases I to III, demonstrating their efficacy against tumors with favorable toxicity profiles. Nonetheless, the increase in dosage limits the drugs due to their dose-dependent toxicities, which include fatigue, nausea, thrombocytopenia, neutropenia, and adverse heart effects. Most importantly, their physical and chemical properties influence blood–brain barrier penetration [70]. These risks may be reduced through the development of brain-penetrant, isoform-selective inhibitors that achieve CNS target engagement at lower systemic doses.

The US Food and Drug Administration has approved several HDACis for the treatment of hematological malignancies, including romidepsin, panobinostat, and belinostat, which target multiple myeloma and cutaneous T-cell lymphoma. Nevertheless, only a handful of HDACs for neurodegenerative disorders have entered clinical trials, while the rest remain at the preclinical stage of development. Despite promising preclinical results, the clinical application of HDACs in neurodegenerative disorders has yet to be achieved [170].

Because of the multi-targeted property of HDAC inhibitors, diverse pathways through which they act, and their interaction with several types of proteins, there is a very low probability of discovering a single mechanism of action of an HDAC inhibitor. Furthermore, the cell’s gene expression, along with the cellular environment, can significantly affect treatment with HDAC inhibitors [171].

Nevertheless, rational combination therapy or carefully controlled multi-target inhibition may recruit complementary mechanisms to overcome the drawbacks of mono-target therapies. A treatment approach that can potentially increase efficacy and reduce undesirable side effects is targeting various molecular targets at once [172,173]. It has been very challenging to develop highly selective HDAC inhibitors owing to the high homology between the zinc-dependent catalytic domains of the various HDAC proteins [174]. The current emphasis is on designing chemical probes with clearly defined molecular targets and superior pharmacokinetic properties so that they can successfully cross the BBB without compromising safety parameters [175].

In depression, direct evidence in humans is still limited, and large-scale randomized controlled trials do not exist. Thus, the clinical importance of HDAC inhibitors is primarily derived from preclinical studies [7], highlighting the critical need for well-designed clinical trials for validating the therapeutic potential and safety of HDACis in depression.

## 7. Future Perspectives for Isoform-Selective HDAC Therapeutics in Depression

Future studies based on dual HDAC inhibitors are expected to focus on generating more selective drugs that precisely target HDAC isoforms like HDAC3 and HDAC6. The aim is to improve therapeutic efficacy and minimize the adverse effects of these drugs. Progress in computational modeling, structure-based drug design, and high-resolution structural techniques will augment zinc-binding group (ZBG) interactions and linker structures. This will ultimately lead to more selective drugs that penetrate the brain and improve the overall pharmacokinetics. At the same time, the increasing use of precision medicine and epigenetic biomarkers may help identify the individuals most likely to benefit from dual HDAC inhibition. Future research would highlight translating preclinical findings into clinical settings to understand better the long-term safety, effectiveness, and neuroprotective qualities of these compounds in neuropsychiatric and neurodegenerative disorders, such as depression. Targeting mechanisms linked to synaptic plasticity, neuroinflammation, and stress-related signaling remains a promising therapeutic approach.

## Figures and Tables

**Table 1:** Classification of histone deacetylase (HDAC) isoforms: functions and their involvement in diseases.

HDAC Class	HDAC Isoforms	Functions	Disease Involvement
Class I	HDAC1	Transcriptional repression, cell proliferation, neurogenesis, synaptic regulation [4], inflammatory regulation [28].	Huntington’s disease [29,30], Amyotrophic lateral sclerosis [31], Alzheimer’s disease [32], Depression [27], Cancer [33]
	HDAC2		Depression [24], Alzheimer’s disease, Schizophrenia [34], Huntington’s disease [35], Cancer [36]
	HDAC3		Depression [37], Alzheimer’s disease [38], Cancer [33], Ischemic stroke [39], Huntington’s disease [40], Multiple sclerosis and demyelinating disease [28]
	HDAC8		Cancer [33], Parasitic infections [41]
Class IIa	HDAC4	Tissue-specific gene regulation [42], synaptic plasticity, memory [43].	Cancer [33], Depression [25], Brachydactyly mental retardation syndrome (BDMR) [44]
	HDAC5		Cancer [33], Depression [25], Stress susceptibility [45]
	HDAC7		Cancer [33], Depression-related behavior, Neuroinflammation and mitochondrial dysfunction [46]
	HDAC9		Cancer [33], Depression [16], Atherosclerosis [47]
Class IIb	HDAC6	Cytoskeletal dynamics, protein trafficking, autophagy, processes relevant to neuronal integrity and neurodegeneration [42].	Cancer [33] Depression, Stress susceptibility and neurodegenerative diseases [48], Parkinson’s disease [49], Huntington’s disease [50]
	HDAC10	DNA damage repair, aging, cell cycle regulation, gene expression [33].	Cancer [33]
Class III Sirtuins	SIRT 1	Stress responses, mitochondrial function, and aging-related pathways. Neuroprotection and metabolic regulation in the brain (SIRT1-3) [51]. DNA damage repair, aging, cell cycle regulation, gene expression [33].	Depression [52], Neurodegenerative disease [53], Cancer [33], Metabolic diseases [54]
	SIRT 2		Depression [26], Cancer [33], Alzheimer’s disease [55], Parkinson’s disease [56]
	SIRT 3		Cancer [33], Depression, Anxiety [57], Cardiovascular diseases [58], Liver fibrosis [59]
	SIRT 4		Cancer [33], Hyperinsulinemia and diabetes, Liver disease, Cancer, Neurodegeneration, Heart disease, Aging, and Pathogenic infections [60]
	SIRT 5		Cancer, Cardiovascular disorders, and Neurodegeneration [61]
	SIRT 6		Cancer [33], Depression [62], Inflammation, Diabetes, Steatohepatitis, Arthritis, Cardiovascular diseases, Neurodegenerative diseases, Viral infections, Renal and corneal injuries [63]
	SIRT 7		Cardiovascular and neurodegenerative diseases, Pulmonary and renal disorders, Inflammatory diseases, Cancer [64]
Class IV	HDAC11	Immune regulation and energy metabolism [51].	Depression, Tumorigenesis, Neuroinflammation, Metabolism [65], Alzheimer’s disease [66], Neuropathic pain [67]

**Table 2:** HDAC3 and HDAC6 inhibitory activities, selectivity profiles, and reported pharmacological effects of selected HDAC inhibitors.

Inhibitor	HDAC3 Activity	HDAC6 Activity	Selectivity Profile	Reported Effect
Compound 24c	IC_50_ = 34 nM	IC_50_ = 2.6 nM	Dual HDAC3/6 inhibitor	Anticancer [11]
RGFP966	IC_50_ = 80 nM	No significant inhibition reported at concentrations up to 15 µM	Selective HDAC3 inhibitor	Antidepressant, Cognitive enhancement [146]
Valproic acid	IC_50_ ≈ 0.7–1 mM	Not reliably reported	Weak inhibitor of class I	Anticancer [147], Antidepressant [148]
Butyric acid/Sodium butyrate	IC_50_ ≈ 135 µM	Not reported	Weak, broad short-chain fatty acid HDAC inhibitor [149]	Antidepressant [150]
Entinostat (MS-275)	IC_50_ ≈ 1.7 µM	Very weak; generally, >20 µM	Class I-selective inhibitor, principally targeting HDAC1 and HDAC3	Anticancer [151,152], Antidepressant [86]
Vorinostat (SAHA)	IC_50_ ≈ 36 nM	IC_50_ ≈ 29 nM	Pan-HDAC hydroxamate inhibitor [153]	Antidepressant [154]
Romidepsin (FK228)	IC_50_ ≈ 1 nM	IC_50_ ≈ 226 nM	Predominantly class I-selective cyclic depsipeptide	Anticancer [153]
Panobinostat (LBH589)	IC_50_ ≈ 2 nM	IC_50_ ≈ 1 nM	Highly potent pan-HDAC hydroxamate inhibitor	Anticancer [153], Neuroprotective [155]
RGFP136	IC_50_ ≈ 0.4 µM	Not reported	HDAC3-preferring benzamide inhibitor; also inhibits HDAC1/2 at higher concentrations	Cognitive enhancement [72]
PT3	IC_50_ ≈ 0.245 µM	IC_50_ ≈ 37.18 μM	Brain-permeable, HDAC3-selective benzamide inhibitor	Cognitive enhancement [89]
Tubastatin A	Not individually reported; approximately 200-fold weaker against class I HDACs	IC_50_ ≈ 18 nM	Selective HDAC6 inhibitor	Antidepressant [98]
ACY-738	IC_50_ ≈ 218 nM	IC_50_ ≈ 1.7 nM	Brain-bioavailable HDAC6-selective inhibitor	Antidepressant [98]
ACY-775	IC_50_ ≈ 11.2 µM	IC_50_ ≈ 7.5 nM	Brain-bioavailable, highly selective HDAC6 inhibitor	Antidepressant [98]
Belinostat (PXD101)	IC_50_ ≈ 19 nM	IC_50_ ≈ 10 nM	Pan-HDAC hydroxamate inhibitor	Anticancer [153]

## Data Availability

No new data were created or analyzed in this study. Data sharing is not applicable to this article.
